# Research on water quality monitoring and abnormal early warning from the dual-driven perspective of knowledge and data

**DOI:** 10.1371/journal.pone.0345405

**Published:** 2026-04-06

**Authors:** Xu Chen, Jiayuan Guo, Ying Zhao, Jingchun Feng

**Affiliations:** 1 Business School, Hohai University, Nanjing, P. R. China; 2 Business School, Hohai University, Nanjing, P. R. China; 3 School of Public Administration, Shandong University of Political Science and Law, Jinan, Shandong, P. R. China; 4 Business School, Hohai University, Nanjing, P. R. China; National Research and Innovation Agency, INDONESIA

## Abstract

Water resources are the foundation for human survival and development, yet intensified human activities have increased the risks of water quality deterioration. Existing water quality early warning studies predominantly emphasize prediction performance while often under-addressing anomaly determination, which contributes to frequent false alarms in operational monitoring. From a dual-driven perspective of knowledge and data, this study develops an integrated framework for water quality monitoring and abnormal early warning. On the knowledge-driven side, abnormal water quality observations are systematically categorized into pseudo anomalies induced by accidental disturbances and true anomalies characterized by sustained structural deviations such as violent fluctuations or upward/downward leaps. Accordingly, reproducible identification strategies are designed, including rolling-statistics-based detection for isolated false data points, rolling-variance screening for variance-shrinkage sequences, and difference-threshold rules for rapid-change segments, enabling the exclusion of pseudo anomalies from model learning and improving alert credibility. On the data-driven side, a Stacking ensemble learning model is constructed by integrating multiple base learners to capture nonlinear temporal patterns and enhance multi-step prediction performance. The proposed framework is validated using high-frequency water quality, water level, and rainfall data from three monitoring stations in the Phase II of the Eastern Route of China’s South-to-North Water Diversion Project. Results show that the ensemble model demonstrates good tracking ability, while the knowledge–data coupling mechanism effectively distinguishes pseudo anomalies from true anomalies, thereby reducing false alarms and improving the practical feasibility of water quality monitoring and early warning.

## 1. Introduction

Water resources are fundamental natural assets and strategic economic resources. The quantity and quality directly affect food production, energy supply, industrial development, ecological conditions and national security [1]. According to the 2024 United Nations World Water Development Report, 2.2 billion people worldwide still lack access to safely managed drinking-water services, indicating persistent and growing pressure on water security. To support water environment governance, monitoring water quality has become a routine practice for understanding environmental conditions and informing management decisions [2]. However, since water conservancy projects are easily damaged by human factors such as industry, the risks during the operation period are greater than those of other projects [3,4]. In large-scale water conservancy and transfer systems, operational risks can be amplified by human-related disturbances such as industrial activities and management complexity, making water quality deterioration and monitoring failures more consequential than in many other types of infrastructure. Therefore, developing reliable approaches to monitor water quality data and implement effective early warning remains an urgent challenge [5].

At present, scholars’ research on water quality early warning models mainly includes two categories, namely physical models and data-driven models. The research on physical models mainly constructs models such as water flow and pollutant diffusion, to achieve early warning. However, their implementation is often more difficult than that of data-driven models and is subject to multiple sources of interference and uncertainty, which has led to data-driven model early warning becoming the mainstream [6].

Data driven early warning can be further classified into two main categories based on its prediction principle: statistical analysis method and prediction method. Statistical analysis method is based on clustering, analyzing and comparing the past data. When certain water quality indicators exceed the limit or show a tendency to exceed the limit, a warning is triggered [7,8]. Since this method usually requires exploring some regularities and patterns of water quality data changes, some literature believes that this method is essentially a knowledge-driven warning model [9,10]. However, this type of early warning model has high calculation and implementation complexity, and the model adaptability is poor in practical applications and across changing conditions [11].

The prediction method uses a series of means, such as traditional time series prediction and artificial intelligence prediction [12], to predict the future evolution of water quality based on the current water quality change law. When the predicted value differs significantly from the actual value, it triggers a water quality early warning. This is a typical data-driven early warning model. Since the prediction method has good tracking performance and can better describe the future trend of water quality changes, it has become the mainstream method for water quality early warning [13].

The early warning of water quality anomalies based on the prediction method is actually divided into two steps: one is the prediction of water quality, and the other is the determination of water quality anomalies. However, research on the determination of water quality anomalies is often neglected. In practice, water quality monitoring and early warning systems developed based on prediction methods often suffer from false alarms, because accidental factors can substantially affect monitoring observations [14,15]. Although in previous studies on prediction methods, attention has also been paid to the impact of abnormal data on early warning systems, such as the water distribution systems developed by Hu et al. [16], which can distinguish the problem of abnormal sensor data; the multi-factor integration water quality anomaly detection algorithm proposed by He et al. [17] has a higher anomaly detection rate and a lower false alarm rate. However, there are many types of water quality anomalies, and there is a lack of classification and exploration of abnormal data. It is necessary to determine whether the data is “pseudo abnormal” caused by accidental factors or true abnormal data.

This paper integrates knowledge-driven and data-driven ideas, constructs a water quality monitoring and abnormal early warning framework to address the practical limitations of prediction-based early warning, especially false alarms caused by accidental disturbances and insufficient attention to anomaly determination. At the knowledge-driven level, the system has established a type-oriented classification for abnormal observations based on temporal characteristics, enabling the identification and exclusion of accidental abnormal data before model learning and thereby improving the credibility of early warning under interferences such as sensor anomalies and rainstorm runoff. At the data-driven level, multiple variables such as rainfall intensity and water level fluctuation are introduced as dynamic correction factors. An integrated prediction model is constructed by combining the advantages of multiple base learners within a Stacking ensemble learning strategy, including SVM, GB, XGB, RF, and KNN, achieving robust capture of nonlinear temporal patterns for multi-step prediction. Through the verification of multiple water quality monitoring points in the Second Phase of the Eastern Route of the South-to-North Water Diversion Project in China, it is found that this model exhibits two complementary functions, namely reliable time-series prediction and improved discrimination between accidental abnormal data and sustained abnormal changes through the knowledge–data coupling mechanism. The research results provide a practical early warning framework for long-distance water transfer projects by jointly improving prediction performance and the credibility of anomaly determination.

## 2. Methodology

### 2.1 Architecture of water quality abnormal early warning model from the dual-driven perspective of knowledge and data

This paper constructs a water quality abnormal early warning model from the dual-driven perspective of knowledge and data. Specifically, a knowledge-driven approach is used in the determination of abnormal water quality, while a data-driven approach is used in water quality prediction (the relevant data is sourced from China South-to-North Water Diversion Group Co., Ltd, and was researched and compiled by the authors).

(1) Knowledge-driven water quality anomaly determination

To avoid the false alarm of water quality anomalies caused by the interference of external factors, based on the analysis of abnormal water quality data, this paper proposes a water quality abnormal early warning method, which classifies abnormal water quality data into “pseudo anomaly” data caused by accidental interference factors such as machines and natural factors, and “true abnormal” water quality data.

(2) Data-driven water quality prediction

There are many water quality prediction models, and scholars often improve the existing models, but the specific performances of different water quality indicators are not the same. Meanwhile, in the detection of abnormal water quality data, a variety of complex environmental factors and parameters are involved, and there may be nonlinear relationships and spatiotemporal changes among these factors [18]. Traditional single models may not be able to fully capture the complex patterns and anomalies in the data, so integration models are more common in water quality prediction [19], such as ensemble learning [20].

**Fig 1:**
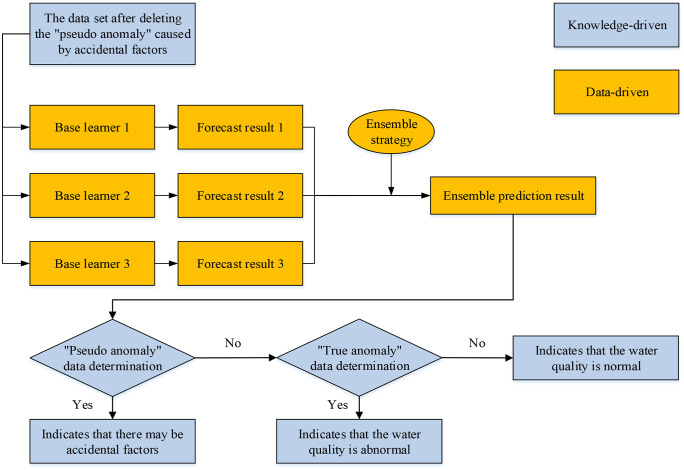
Model architecture diagram.

In summary, the schematic diagram of the water quality abnormal early warning model from the dual-driven perspective of knowledge and data described in this paper is shown in Fig 1. In this framework, the knowledge-driven component focuses on anomaly-type determination and the exclusion of accidental abnormal observations before model learning, while the data-driven component focuses on time-series prediction and residual-based warning generation.

### 2.2 Analysis of abnormal water quality data driven by knowledge

In statistics, data anomalies can be understood as: not following a specific data distribution law and being far away from other normal points; in data mining, data anomalies can be understood as: data that deviates significantly from datasets of the same category in data classification. Based on the above definitions, abnormal water quality data actually contains two meanings [21]. One is the data that is significantly different from other data series, which is the potential premise of most studies, and the other is abnormal data, which refers to untrue data in the time dimension, which includes some anomalies caused by accidental errors. However, there is still an overlap in concepts of these two types of abnormal data. For example, anomalies caused by sensor errors often exhibit characteristics that are not the same as those of other data series in data.

And further refer to the relevant research of Ma et al [22], this paper classifies abnormal water quality data into the following categories: the first category is false data points, which are usually a point outside the normal range; and the second category is sequence anomaly, that is, the water quality data shows a different fluctuation state from the past within a certain period of time. This paper deeply analyzes the data sent back by some potassium permanganate sensors in the Yangtze River Basin of China and finds that both types of anomalies have their specific manifestations. The specific examples are analyzed as follows.

#### 2.2.1 Abnormal false data points.

**Fig 2:**
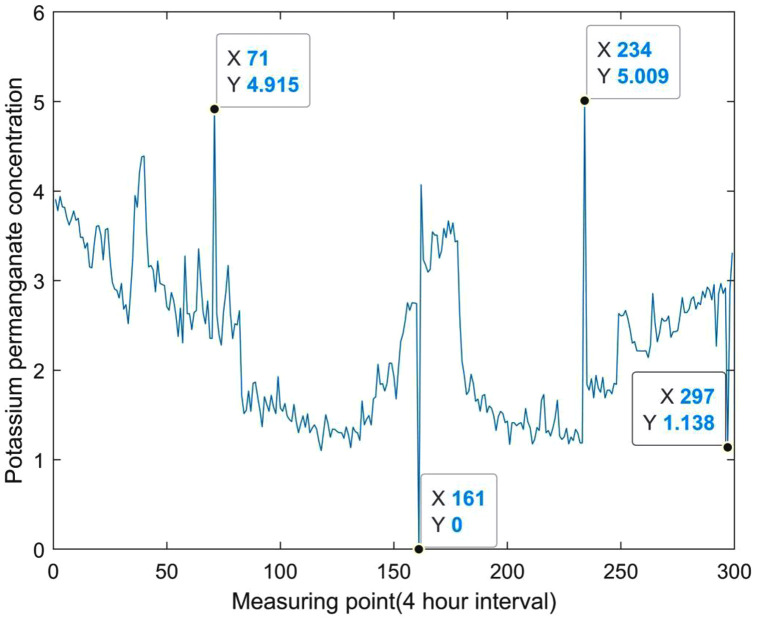
Schematic diagram of abnormal false data points.

It means that in a set of fluctuating data, a peak value or valley value suddenly appears, and the subsequent measurement data returns to normal. This type of anomalies is mostly caused by sensor or data transmission failures. An example is shown in Fig 2.

As shown in Fig 2, there are 4 abnormal false data points, which are significantly higher or lower than other values, and the data does not continue a certain time series. According to the research of Thu et al. [23], this type of data anomaly often stems from accidental factors, so this type of data anomaly needs to be excluded from the early warning system and belongs to “pseudo anomaly” data.

#### 2.2.2 Sequence anomaly.

Sequence anomaly means that the changes of water quality are abnormal within a period of time, including three specific manifestations.

(1) The variance is too small

**Fig 3:**
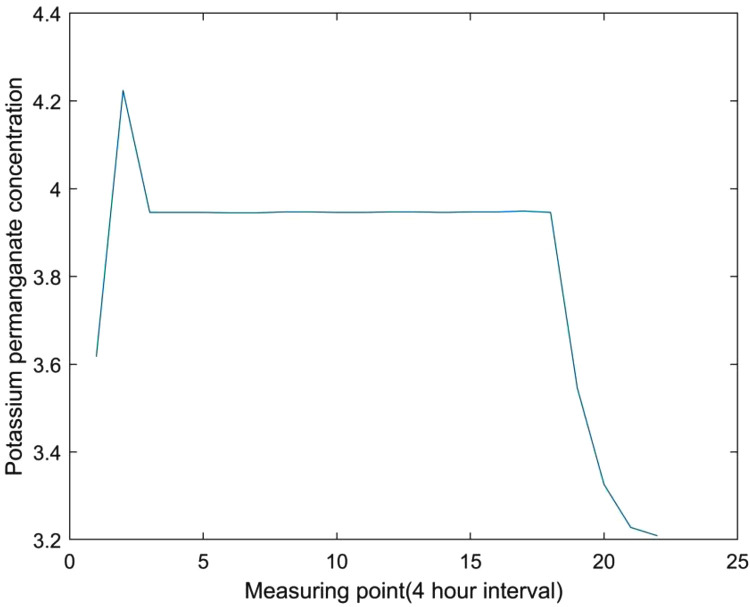
Schematic diagram of abnormal sequences with too small variance.

When the variance among four consecutive measurement points is less than the threshold, it can be determined that the variance is too small. The specific manifestations of this type of data anomalies are shown in Fig 3.

As shown in Fig 3, the concentration of potassium permanganate remains basically stable during a relatively long measurement period, while the data before and after it shows normal fluctuations. This type of data anomaly also needs to be excluded in the early warning system and is classified as “pseudo anomaly” data.

(2) Violent fluctuation

**Fig 4:**
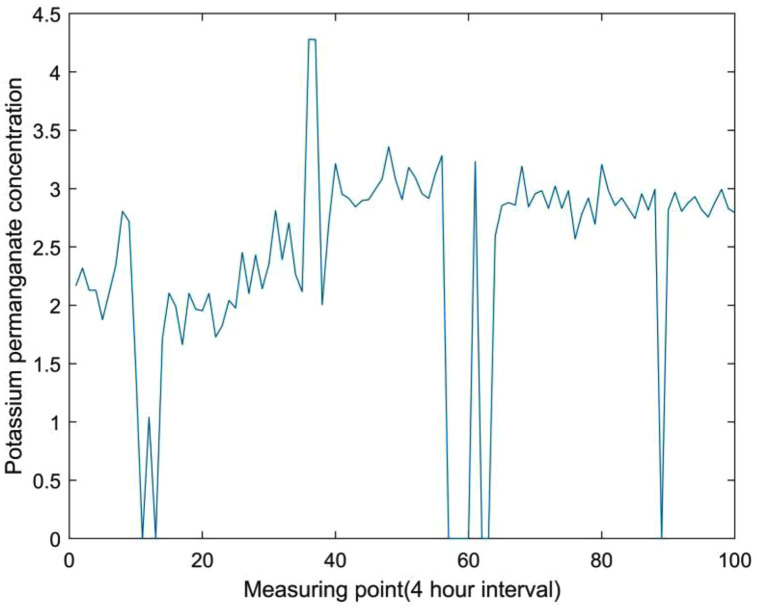
Schematic diagram of the abnormal sequence of violent fluctuations.

This type of anomaly is usually manifested as violent fluctuations in data, which may be caused by weather conditions or human intervention. Regardless of the cause, they need to be taken seriously and marked as abnormal data for further confirmation. The schematic diagram is shown in Fig 4.

(3) Upward or downward leap

**Fig 5:**
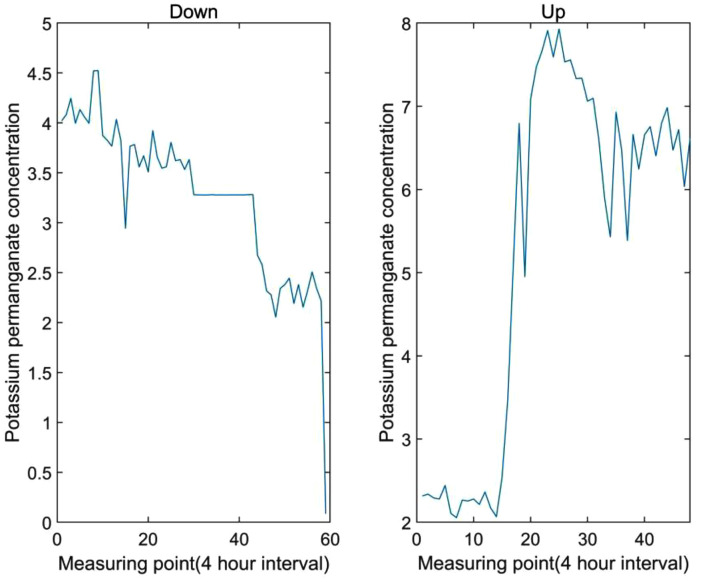
Schematic diagram of abnormal sequence of upward or downward leap.

Unlike violent fluctuations, this type of data anomalies, after showing a trend, cannot recover in a short time but continue to move upward or downward. This type of anomalies often mean that there are indeed abnormal problems in water quality and need to be marked as key points. The schematic diagram is shown in Fig 5.

To sum up, water quality data may be affected by various accidental factors, which may make water quality maintenance and management based on the data ineffective. In addition to removing abnormal data caused by accidental factors from the training samples of the prediction model, it is also necessary to effectively identify in the prediction results which abnormal data is “true anomaly” data to avoid false alarms.

To facilitate reproducibility, each anomaly type is linked to an operational criterion used in Section 2.3, including rolling-statistics deviation for isolated false points, rolling-variance screening for variance-shrinkage sequences, and difference-threshold screening for rapid-change segments.

### 2.3 Abnormal data identification

#### 2.3.1 Rationale and definitions.

In prediction-based early warning, abnormality detection involves not only water quality prediction but also anomaly determination. In practice, monitoring observations can be affected by accidental disturbances such as sensor or transmission anomalies and short-lived hydrological shocks, which often lead to false alarms. To enhance the credibility of early warning, this section develops a knowledge-driven identification procedure to distinguish accidental abnormal observations from sustained abnormal changes in water quality.

According to the temporal characteristics described in Section 2.2, accidental abnormal observations are treated as pseudo abnormal data, while sustained abnormal changes are treated as true abnormal data candidates. The identification rules are designed to be reproducible and are directly linked to measurable time-series characteristics.

#### 2.3.2 Parameter settings.

To ensure reproducibility, the key parameters used in the knowledge-driven identification are summarized in Table 1. Parameter values are determined according to the monitoring frequency and station-specific data characteristics and remain fixed during model training and evaluation.

**Table 1:** Key parameters.

Parameter	Symbol	Meaning	How it is set in this study	Used for
Rolling window length	w	Number of observations in the rolling window	Set according to the 4-hour sampling interval and the short-term disturbance scale described in the monitoring data	Rolling mean and rolling standard deviation, rolling variance
Std multiplier	k	Sensitivity coefficient for deviation from rolling statistics	Empirically set to control the sensitivity of isolated false-point detection	Isolated spike or valley detection
Variance threshold	ε	Threshold for variance shrinkage within a rolling window	Empirically set to identify near-constant segments indicative of transmission or sensor stagnation	Variance shrinkage sequence detection
Difference threshold	Δ_th	Threshold for first-order difference magnitude	Empirically set based on the magnitude of abrupt changes observed in the monitored series	Violent fluctuation and leap detection
Residual threshold	R_th	Triggering threshold for residual-based abnormal flagging	Set as two times the RMSE of the corresponding prediction model	Initial abnormal flagging before type screening
Consecutive rule	n	Minimum consecutive times to confirm a persistent abnormal segment	Set to 2 to reduce single-point false alarms as indicated by the empirical observation in this study	Persistent abnormal confirmation

#### 2.3.3 Rule identification.

(1) Rule 1 Identification of isolated false data points using rolling statistics

Isolated false points usually appear as short-lived spikes or valleys that do not reflect the actual evolution of water quality. Let $x_{t\;}$ denote the observed water quality indicator at time t. For each time t, a rolling window of length w is constructed to compute the rolling mean $\mu_{t}$ and rolling standard deviation $\sigma_{t}$. A point is marked as an isolated false point when its deviation from the rolling center exceeds the rolling dispersion:


$$|x_{t\;}-\mu_{t}|>k\cdot \sigma_{t}\;$$ (1)

Points satisfying this rule are classified as pseudo abnormal data, because they are consistent with accidental disturbances and are not expected to persist in subsequent observations.

(2) Rule 2 Identification of variance-shrinkage sequences using rolling variance

Variance shrinkage sequences are characterized by abnormally small fluctuations over a period, which may be caused by sensor stagnation, transmission faults, or other accidental factors. For each rolling window, the rolling variance $Var_{t}$ is computed. A segment is flagged as a variance-shrinkage sequence if:


$$Var_{t}\;<\varepsilon$$ (2)

All observations within such flagged windows are classified as pseudo abnormal data, because the sequence reflects an abnormal measurement process rather than genuine water quality dynamics.

(3) Rule 3 Identification of rapid-change segments using difference thresholds

True abnormal changes in water quality often manifest as violent fluctuations or upward and downward leaps, which are reflected by abrupt changes between consecutive observations. The first-order difference is defined as:


$$d_{t}\;=x_{t}\;-x_{t-1}\;$$ (3)

A rapid-change segment is flagged when:


$$|d_{t}|>\Delta_{th}\;$$ (4)

Observations associated with rapid-change segments are treated as true abnormal data candidates, because they reflect sustained structural deviation patterns rather than single-point accidental disturbances. To reduce false alarms caused by isolated abrupt points, the consecutive rule is applied: only segments satisfying the above condition for at least n consecutive times are confirmed as persistent abnormal candidates.

#### 2.3.4 Integration with prediction-based residual triggering.

The knowledge-driven identification is coupled with the residual-based triggering mechanism in prediction-based early warning. Let ${\hat{x}}_{t}$ denote the predicted value from the data-driven model at time t, and let the residual be $e_{t}=x_{t}-{\hat{x}}_{t}$.A point is first flagged as abnormal when:


$$|e_{t}|>R_{th}\;$$ (5)

where $R_{th}$ is set to two times the RMSE of the corresponding prediction model. The flagged points are then processed by the above knowledge-driven rules to determine whether they are pseudo abnormal data or true abnormal candidates. This coupling mechanism reduces false alarms by excluding accidental disturbances before early warning decisions.

#### 2.3.5 Algorithmic workflow.

Algorithm 1 summarizes the complete knowledge-driven identification procedure. The workflow outputs two sets: pseudo abnormal data to be excluded from model learning and early warning, and true abnormal candidates to be retained for subsequent early warning decisions.

**Algorithm 1. Knowledge-driven abnormal data identification workflow**

Input: observed series $x_{t}$,predicted series ${\hat{x}}_{t}$,window length w, parameters $k,\varepsilon ,\Delta_{th}\;$,residual threshold $R_{th}$,consecutive rule n.

Output: pseudo abnormal set P,true abnormal candidate set T

1.Initialize P=emptyset,T=emptyset

2.For each time t, compute residual $e_{t}=x_{t}-{\hat{x}}_{t}$

3.If $|e_{t}|\leq R_{th}\;$,continue to next time t

4.Compute rolling statistics $\mu_{t},\sigma_{t}$ using window length w

5.If $\left|x_{t}-\mu_{t}\right|>\sigma_{t}\cdot k$ add $x_{t}$ to P and continue

6.Compute rolling variance $Var_{t}$ using window length w

7.If $Var_{t}\;<\varepsilon$ add all points in the corresponding window to P and continue

8.Compute difference $d_{t}\;=x_{t}\;-x_{t-1}\;$

9.If $|d_{t}|>\Delta_{th}\;$ holds for at least n consecutive times, add involved points to T otherwise treat the isolated point as pseudo abnormal and add to P

10.Return P and T

The above rules are designed to be transparent and reproducible by explicitly linking each abnormal type to an operational time-series criterion. Parameter values can be calibrated for different stations and basins based on monitoring frequency and data characteristics, while the rule structure remains unchanged. This design supports practical deployment in operational monitoring settings where accidental disturbances are common.

### 2.4 Data-driven ensemble learning for water quality prediction

#### 2.4.1 Machine learning model definitions.

Machine learning models have been increasingly used in sensor-driven monitoring and early warning for complex networked infrastructures (e.g., natural gas pipelines and integrated energy systems), where data are typically nonlinear, time-dependent, and subject to regime shifts and uncertainty. Prior studies show that deep sequence models are commonly implemented for short-term forecasting by combining multi-scale signal processing and recurrent architectures. For instance, wavelet transform has been integrated with an enhanced Deep-RNN to capture multi-resolution temporal patterns and improve hourly demand prediction accuracy [24]. Deep learning has also been adopted for dynamic prediction of key operational properties in gas systems, illustrating practical implementations based on sliding-window inputs and supervised learning to model time-lag effects [25].

Beyond point prediction, probabilistic deep learning and state-switching models have been employed to represent uncertainty and operational regimes. A representative implementation integrates probabilistic deep learning with a Gaussian mixture model and a hidden Markov model (GMM–HMM) for data-driven reliability assessment, enabling regime-aware inference under stochastic conditions [26]. Related work further demonstrates data-driven pipelines for anomaly detection and vulnerability dynamic analysis in large-scale integrated energy systems, supporting early identification of abnormal states and evolving vulnerabilities [27]. In resilience-oriented applications, uncertainty analysis has been incorporated to evaluate pipeline-system resilience under uncertain disturbances, which is important for robust decision support [28].

For operational decision-making, reinforcement learning and heuristic optimization provide implementation routes that connect prediction with management actions. Deep reinforcement learning has been used for predictive management of demand response in natural gas pipeline networks, enabling adaptive policies learned from system feedback [29]. Heuristic optimization methods such as particle swarm optimization (PSO) have been applied to emergency dispatch optimization in pipeline networks, offering a computationally tractable approach for constrained decision problems [30].

In terms of deployability, studies also emphasize hyperparameter optimization and knowledge mining. Bayesian optimization combined with XGBoost has been reported for fault early warning in ultrasonic flowmeters, illustrating automated tuning for improved generalization [31]. Interpretable combinatorial ML frameworks that integrate XGBoost with optimization strategies have been used to improve transparency while maintaining prediction performance [32]. In addition, large language models (LLMs) have been applied to knowledge mining from fault text data and reliability contexts, supporting structured knowledge extraction for diagnostic decision support [33,34]. Similar data-driven implementations have also been reported for nonlinear property prediction in oil-related engineering tasks using deep learning and combinatorial ML models [35,36].

Motivated by these established implementations, the present study adopts an ensemble-learning workflow for water-quality prediction and early warning, which is compatible with robust model training/screening and subsequent Bagging, Boosting, Stacking integration described in the following subsections.

#### 2.4.2 Training and screening of base learners.

This paper needs to make early warnings based on water quality data, which is inseparable from water quality prediction [37]. According to the research of Zhu et al. [38], the main models for predicting water quality data are time series dynamic prediction models, such as machine learning models based on time series [39]. In the theoretical structure of machine learning, prediction is a very important part. By learning the temporal patterns of water quality data, machine learning models can support time series prediction and provide a basis for subsequent early warning. However, relying solely on a single machine learning model may lead to the instability of learning ability. An excessively high learning ability may cause overfitting problems, while insufficient learning ability may result in underfitting, which may cause errors in the prediction and warning decision of water quality changes under operational disturbances. To improve the stability and effectiveness of machine learning, some scholars have proposed the idea of ensemble learning [40,41].

Ensemble learning is an algorithmic structure that creates an outstanding ensemble learning model by combining multiple simple machine learning model strategies [42,43]. This approach can effectively reduce the errors caused by the limitations of a single learner and can effectively deal with noise and emergencies in the data. This technology is particularly important for the frequently changing environmental factors in water quality monitoring, and can promptly detect and respond to abnormal situations in the constantly updated data stream. Compared with heavier deep sequence models, the ensemble strategy adopted in this paper provides clearer training and deployment procedures and lower computational cost while maintaining stable tracking performance for high frequency operational monitoring data.

In order to implement the ensemble learning model, it is necessary to select an appropriate base learner. Firstly, based on the characteristics of the water quality data studied and other data generated during the operation of water conservancy projects, the base learners are initially screened; by performing random and replayed sampling operations on the sample dataset, sub-training datasets with certain differences are obtained; the sub-training datasets are adopted to train and test the base learners, and the models of the base learners are constructed by adjusting relevant parameters or functions. By comparing the performance of different models on the test dataset and combining the concept of ensemble learning, the base learners are screened. When constructing an ensemble learning model, the training and selection of learners are particularly crucial.

This paper selects the Support Vector Machine (SVM) Model, Gradient Boosting (GB) Model, Extreme Gradient Boosting (XGB) Model, Random Forest (RF) Model, Logistic Regression (LR) Model, and K-Nearest Neighbors (KNN) Model as base learners to learn the data, and then they are screened according to the test results. In this study, the final base learners used for ensemble learning are determined after performance screening and residual diagnostics, and the logistic regression learner is excluded due to residual autocorrelation indicated by the Durbin Watson statistic, while SVM, GB, XGB, RF, and KNN are retained as base learners.

#### 2.4.3 Ensemble strategy construction.

The output data of the base learner is used as the input data of the ensemble learning model, and then ensemble learning is conducted on the input data according to different strategies to obtain the corresponding output results. By comparing the output results of different strategies with the actual labels of the samples, the performance of the ensemble learning models of different strategies is analyzed, and the strategy with better generalization ability is selected as the final strategy of the ensemble learning, thereby constructing an ensemble learning model for abnormal monitoring of water quality data. In this paper, three ensemble strategies, namely Bagging, Boosting, and Stacking, are selected. The prediction performance of the ensemble models under each strategy is compared through cross-validation to determine the final optimal ensemble strategy. For the Stacking strategy, the predictions of the screened base learners constitute the second level input features, and a meta model is trained to integrate the base learner outputs and produce the final prediction, thereby improving generalization and stabilizing the prediction under complex disturbances.

## 3. Case study

### 3.1 Case overview

The South-to-North Water Diversion Project in China is a grand water conservancy project and an inter-basin and inter-regional strategic infrastructure, which aims to optimize the water allocation pattern in China and solve the problem of water shortage in the north, covering the Yellow River, Huai River, Hai River and regions such as ‌Beijing, Tianjin, Hebei, Henan, Shandong, etc. Among them, the Second Phase of the Eastern Route of the South-to-North Water Diversion Project is an important part, aiming to further optimize the water resource allocation in North China, improve water supply capacity, and promote the sustainable development of the regional economy.

The total length of the Second Phase of the Eastern Route of the South-to-North Water Diversion Project in China is 4,278.1 kilometers. It is arranged along the urban development axis of the Taihu Lake-Hangzhou Bay urban agglomeration and the coastal areas of the Yellow Sea. The starting point is located at a certain reservoir in the Taihu Lake Basin of Jiangsu Province, passing through cities such as Suzhou, Jiaxing, and Zhoushan, and finally entering the East China Sea. This will greatly enhance the water transmission capacity of the Eastern Route Project and meet the water demand of the cities along the route. The project will expand and build multiple reservoirs, such as Hengshui Lake Reservoir, Dalangdian Reservoir, Beidagang Reservoir, etc., as well as 51 new pumping stations including Guangling Station and Gaoyou Station, with an additional installed capacity of 1.32 million kw. The target for the popularization rate of water-saving appliances is 98% to 100%, the leakage rate of urban water supply pipelines is controlled at 8% to 10%, and the reuse rate of industrial water should reach 90% to 98%.

However, in such an important water conservancy project, the monitoring and early warning of water quality are still problems. In addition to the high complexity of water quality monitoring in the water source area itself, the insufficient overall information sharing capacity and intelligent scheduling, deployment and precise control capabilities are also important reasons.

In order to verify the effect of the water quality monitoring and abnormal early warning model from the dual-driven perspective of knowledge and data described in this paper, the data collected from the water quality stations of Baoying(33.24°N, 119.36°E‌), Linjiaba(34.25°N, 117.20°E‌) and Longhekou(31.50°N, 117.00°E‌) in the Second Phase of the Eastern Route of the South-to-North Water Diversion Project is selected as research cases.

To address the applicability of the proposed framework beyond the three stations, this case study is designed as a station-level verification under a long-distance transfer project. The framework can be extended to larger water transfer networks by replicating the same data stream acquisition and model deployment procedure at additional stations, while key parameters and model components may require station-specific calibration and further cross-station testing.

### 3.2 Data collection and processing

(1) Data collection

This paper samples three water quality stations along the Second Phase of the Eastern Route of the South-to-North Water Diversion Project in China and systematically collects and processes the data from January 1, 2024 to December 31, 2024. The collected data is collected by sensors and uploaded every four hours. The three stations are three sets of data, mainly including water quality, water level and rainfall data. The water quality data covers several key water quality indicators such as temperature, PH value, dissolved oxygen, potassium permanganate, Nephelometric Turbidity Unit (NTU), total phosphorus, conductivity (μs/cm), etc. The water level data includes information such as upper water level, lower water level, flow rate etc. The rainfall data refers to the cumulative rainfall (unit: millimeters) and the current ten-minute rainfall. Ultimately, a total of 2,190 real-time monitoring data is collected.

Because the original sensor data are subject to preprocessing and quality control, the final modeling sequence may not strictly retain a complete record at every four-hour interval. This is consistent with the operational data processing setting and is explicitly considered in subsequent modeling and evaluation.

Regarding sensor calibration and maintenance information, the monitoring data used in this study are owned and managed by a third-party operator. Detailed calibration protocols and maintenance logs are not available to the authors under the data access restrictions. To mitigate the potential influence of accidental disturbances related to sensors or transmission, the subsequent knowledge-driven component explicitly targets isolated spikes, variance-shrinkage segments, and rapid-change points during anomaly determination.

(2) Dimensionless processing

**Fig 6:**
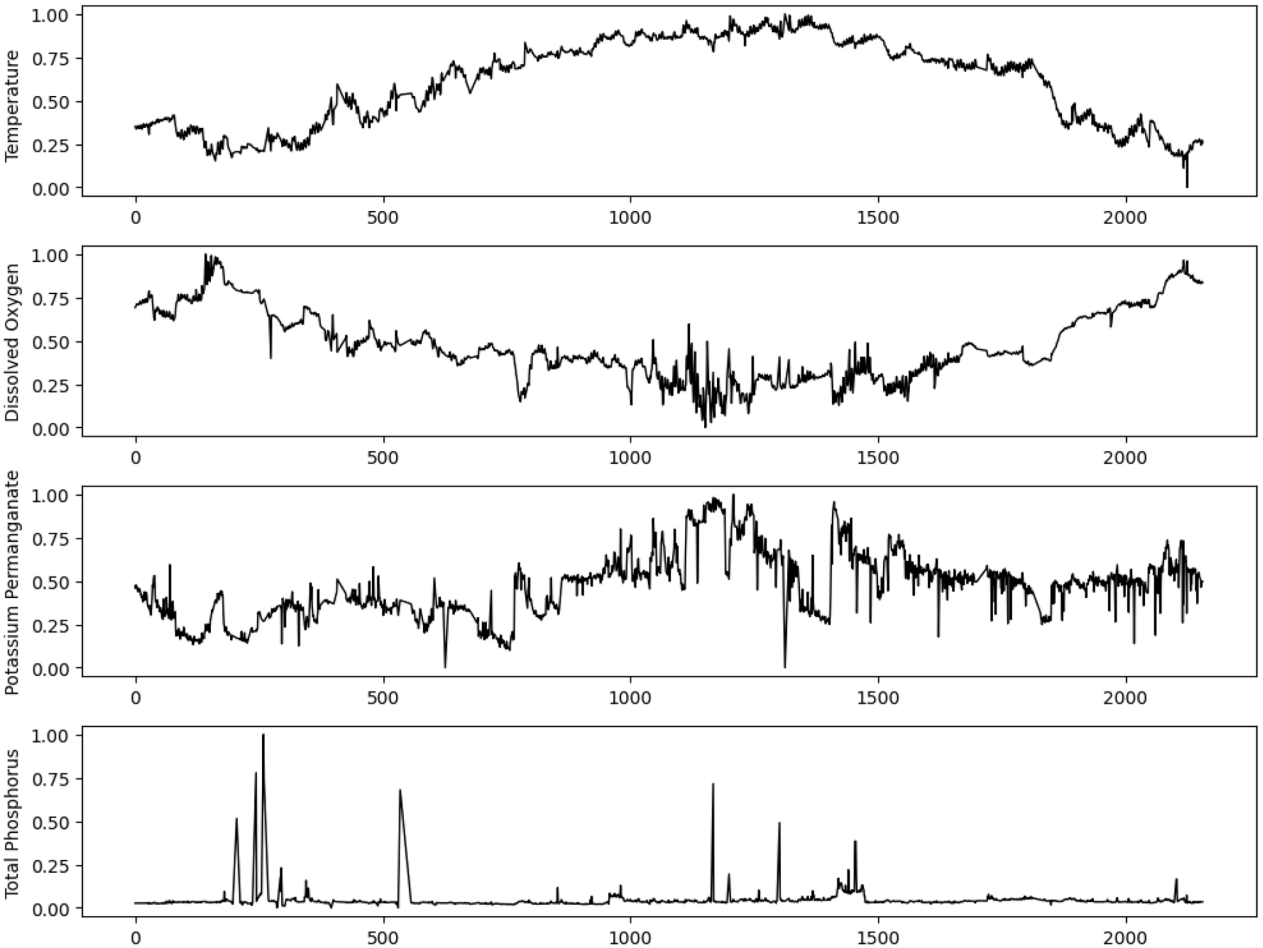
Dimensionless water quality data.

In order to uniformly measure the data of various water qualities, it is necessary to preprocess the original data first. Since each water quality parameter has a different dimension, the data needs to be dimensionless. This paper uses the normalization method to process the water quality data and obtain a dimensionless data sequence with a mean of 0 and a variance of 1. The processed water quality data is shown in Fig 6.

As shown in Fig 6, the horizontal axis represents the time axis, and the vertical axis represents the standardized water quality parameter values. It can be seen from the figure that after data standardization, the data sequence is concentrated in the [−1,1] interval, which is conducive to further data analysis.

Data access restriction statement for this case study: the data cannot be shared publicly because they are obtained from China South to North Water Diversion Group Co., Ltd., and the authors do not have permission to share them with others.

### 3.3 Base learners and ensemble strategy selection

(1) Base learner screening

The method described in Section 2.3 is used to mark and remove the abnormal data in the data. Six base learners are used to learn the data samples collected from Baoying and Linjiaba Water Quality Stations, respectively. The learning effects are shown in Table 1. As noted in Section 3.2, because the original monitoring data are subject to preprocessing and quality control, the final modeling sequence may not strictly retain a complete record at every four-hour interval, and the subsequent learning and evaluation are performed on the preprocessed sequence.

Table 2 shows that under the same training and evaluation setting, the performance of the preselected base learners varies, but the performance parameters of the logistic regression model are significantly worse than those of other learners. After calculation, it is found that the Durbin-Watson Statistic is 0.912, close to 0, and there is a positive autocorrelation, indicating that the model residuals are not independent, which violates one of the assumptions of the linear regression model, so the LR Model should not be accepted as the base learner. After excluding the logistic regression model, SVM Model, GB Model, XGB Model, RF Model and KNN Model are selected as the base learners of the ensemble learning model. The above five learners have excellent performance, diverse principles, and different prediction results, which meet the requirements of the ensemble learning model.

**Table 2:** Learning effects of the base learners.

Model name	Evaluation indicators of learning effects			
	MSE	R-squared	MAE	RMSE
SVM Model	0.0894	0.8459	0.1137	0.2548
GB Model	0.0161	0.9723	0.1137	0.2548
XGB Model	0.0064	0.9890	0.1137	0.2548
RF Model	0.0057	0.9902	0.3546	0.4790
LR Model	0.2295	0.6047	0.3546	0.4790
KNN Model	0.0649	0.8882	0.1137	0.2548

(2) Ensemble strategy selection

After the base learner screening is completed, the results of the three ensemble strategies are verified by the data collected at Longhekou Water Quality Station. The prediction effects of different ensemble strategies are verified respectively from the four perspectives of MSE, R-squared, MAE, and RMSE of water quality prediction. The performance indicators of the three basic strategies are shown in Table 3.

**Table 3:** Evaluation results of the three ensemble strategies.

Ensemble strategy	MSE	R-squared	MAE	RMSE	Training time
Bagging	0.0161	0.9723	0.0750	0.1269	7.5860
Boosting	0.0212	0.9635	0.0814	0.1456	11.3880
Stacking	0.0073	0.9874	0.1137	0.2548	1.4700

Comparing the training results of the three strategies, it is found that the MSE indicator of Stacking is closer to 0 than the other two, and the R-squared indicator is closer to 1 than the other two strategies. It can be seen that the generalization ability of Stacking ensemble strategy is stronger. Comparing Table 1, the learning results of the five base learners are reviewed. The MSE of the ensemble learning Stacking model is smaller than the indicator of the base learners, and the R-squared is larger than the indicator of the base learners, indicating a stronger generalization ability. In conclusion, Stacking is selected as the ensemble learning strategy.

**Fig 7:**
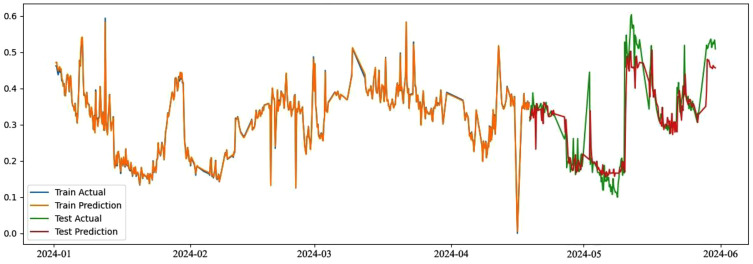
Comparison of deviations between predicted and actual values.

Since the data have been preprocessed, the time series used for modeling may not strictly follow a fixed sampling interval, as described in Section 3.2. Taking the potassium permanganate data collected by Baoying Water Quality Station as an example, a total of 500 sets of data from January 1, 2024 to April 17, 2024 are selected to train the ensemble learning prediction model shown in this paper. A total of 200 sets of data from April 18, 2024 to June 1, 2024 are used as controls to compare the deviation between the predicted values and the actual values. The prediction residuals of the data sequence are obtained, as shown in Fig 7.

It can be seen from Fig 7 that the water quality data prediction based on Stacking ensemble strategy has good tracking characteristics in the training dataset, can effectively track the water quality data, the predicted values are relatively close to the actual values, and the fitting effect is good, indicating the good tracking performance of Stacking ensemble model. On the test dataset, the MSE is 0.0032, which is still a good result. Therefore, it can be considered that Stacking ensemble model has excellent anomaly detection and tracking characteristics.

### 3.4 Water quality early warning effect

**Fig 8:**
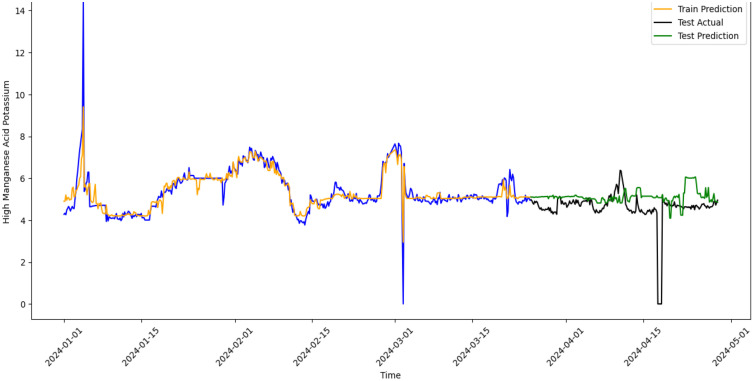
Water quality prediction results of Longhekou Water Quality Station.

In order to verify the effectiveness of the water quality monitoring and abnormal early warning model from the dual-driven perspective of knowledge and data described in this paper, taking the potassium permanganate water quality data observed at Longhekou Water Quality Station as an example, a total of 500 sets of data from January 1, 2024 to March 26, 2024 are selected to train the model, and a total of 200 sets of data from March 27, 2024 to April 29, 2024 are used as test samples. According to the content of Section 2.2, the abnormal points in the training set are removed and then trained, and the water quality of the test dataset is predicted. The results are shown in Fig 8.

As shown in Fig 8, the predicted values of both the training dataset and the test dataset are somewhat different from the actual values. The reason is that the abnormal data is removed in the training dataset.

Then the water quality anomalies are marked. Firstly, the deviation between the predicted values and the actual values is compared to obtain the prediction residuals of the data sequence, and the root mean squared error (RMSE) between the prediction results in the training set and the test set and the actual observed values is calculated respectively, which serves as the overall prediction error evaluation indicator of the model on their respective datasets. If the difference between the predicted result and the true observed value is greater than 2 times the RMSE, the data point is first marked as “abnormal” for further distinction; otherwise, it is “normal”. Some of the abnormal points marked in the data are shown in Table 4.

**Table 4:** Some of the abnormal points marked in the data.

Time	Actual value	Predicted value	Deviation	Dataset
2024-01-03 16:00:00	3.1430	1.9615	1.1815	Training dataset
2024-01-07 08:00:00	4.3830	3.2655	1.1175	Training dataset
2024-01-07 12:00:00	4.3900	3.2237	1.1663	Training dataset
2024-01-12 16:00:00	4.9150	3.3656	1.5494	Training dataset
2024-01-14 16:00:00	1.7150	2.6327	0.9177	Training dataset
2024-01-14 20:00:00	1.5150	2.4820	0.9670	Training dataset
2024-01-15 00:00:00	1.5520	2.4398	0.8878	Training dataset
2024-04-15 20:00:00	0.0000	3.6665	3.6665	Test dataset
2024-04-16 00:00:00	0.0000	3.8820	3.8820	Test dataset
2024-04-16 04:00:00	0.0000	3.8819	3.8819	Test dataset
2024-04-16 12:00:00	0.0000	3.5131	3.5131	Test dataset
2024-04-16 16:00:00	0.0000	3.5487	3.5487	Test dataset
2024-04-21 00:00:00	0.0000	4.0288	4.0288	Test dataset
2024-04-23 08:00:00	0.4880	3.9585	3.4705	Test dataset

To align the case-study evaluation with the purpose of early warning, the warning outcome in this section is determined by the residual-triggering rule above and the subsequent type determination described in Section 2.3, rather than by prediction accuracy alone. In this setting, a false alarm corresponds to abnormal points that are finally determined as pseudo anomaly, while an effective warning corresponds to abnormal points that are finally determined as true anomaly candidates.

After identifying the abnormal points with significant prediction residuals, this paper further conducts a deeper type determination on the abnormal points based on the knowledge-data dual-driven model structure shown in Fig 1 and the classification basis of water quality anomalies in Section 2.2 to distinguish between “pseudo anomaly” and “true anomaly”. Specifically, if the observed value shows isolated peaks in the time series (for example, only one-point deviates significantly from the previous and subsequent trends), it may be a false data point and is determined as a “pseudo anomaly”. If there are violent fluctuations, trend leaps, or significant variance shrinkage before and after the abnormal point, it is more likely to be a structural change and is determined as a “true anomaly”.

Based on the determination principle, this paper further designs a set of automatic marking system based on local fluctuation intensity to further classify the abnormal points. Specifically, the system estimates the local standard deviation of the deviation sequence in a sliding window manner. When the prediction residual of an abnormal point not only significantly exceeds the global error threshold, but also shows higher volatility than its neighboring points, this data point is determined as a “true anomaly”. After testing, it is found that when abnormal points appear 2 times in a row (i.e., the monitoring values are abnormal for 4 consecutive hours) or more, the effect is better, indicating that there may be structural problems such as systemic problems and changes in water quality levels in the interval; otherwise, it is regarded as a “pseudo anomaly” that may be caused by sensor noise or accidental disturbances.

**Fig 9:**
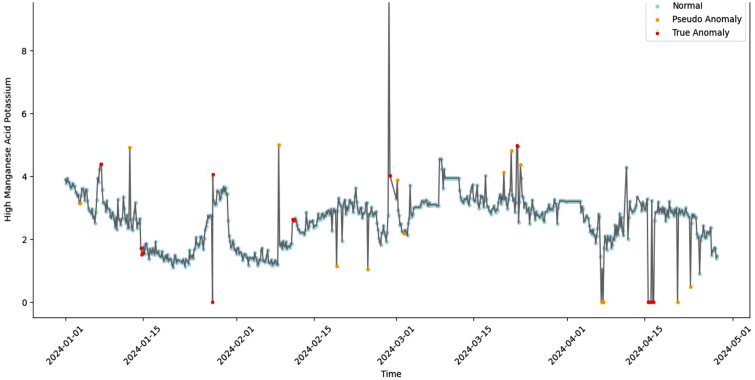
Abnormal water quality markings at Longhekou Water Quality Station.

The automated strategy in this paper combines the residual intensity with the structural characteristics of the time series to achieve dynamic discrimination of abnormal types. Moreover, the determination results are visually annotated through graphical means, enhancing the model’s ability to interpret complex water quality changes and its application value. The specific marking results are shown in Fig 9.

For reporting early warning performance in a way that is comparable across studies, the warning results can be summarized using precision-recall style indicators based on the final categories. Specifically, precision can be defined as the proportion of points marked as abnormal that are finally determined as true anomaly candidates, and the false-alarm rate can be reflected by the proportion of pseudo anomaly points among all abnormal points. These indicators are computed from the final marking results without requiring additional external labels, which is suitable for operational data under access restrictions.

As shown in Fig 9, all the data is marked into three categories, namely normal data, pseudo abnormal data, and true anormal data. The pseudo abnormal data contains most of the distorted data points and data sequences with too small variance, indicating that the water quality monitoring and abnormal early warning model under the knowledge-data dual-driven perspective described in this paper can effectively distinguish the abnormal water quality data.

## 4. Discussion

Water quality monitoring and early warning play a significant role in the sustainable development of a country [1]. For a long time, the field of water quality early warning has attached great importance to prediction technology. Scholars have focused more on the prediction work in early warning [13], and are committed to predicting water quality data more accurately [12], but have ignored the important prerequisite that the purpose of prediction is to provide early warning. This paper integrates the knowledge-driven water quality early warning model into the data-driven water quality early warning model, forming a water quality abnormal early warning model from the knowledge-data-driven perspective. Compared with the prediction model of He et al. [17], it focuses on supplementing the analysis of abnormal water quality categories, elaborates the specific manifestations of “true anomaly” and “pseudo anomaly” in water quality anomalies, and makes the identification of abnormal water quality more accurate and more interpretable for operational decision-making.

This paper selects the data collected from the water quality stations of Baoying, Linjiaba and Longhekou in the Second Phase of the Eastern Route of the South-to-North Water Diversion Project in China as specific research cases. After testing, in terms of prediction performance, the prediction model constructed based on ensemble learning in this paper has a strong generalization ability and can achieve multi-step prediction with high accuracy(Figs 7 and 8), but its accuracy is still slightly lower than that of mainstream research [14,15]. The reasons lie in that, first, some abnormal data is eliminated in the training samples, and second, more attention is paid to the generalization ability in the model training to support stable early warning under disturbances. Under the same data acquisition protocol, the station-level verification provides a practical basis for deploying the framework at additional stations along the transfer corridor, while station-specific calibration and cross-station testing are still necessary.

In terms of abnormal water quality early warning, this paper combines the residual intensity with the structural characteristics of the time series through an automated strategy to achieve dynamic discrimination of abnormal types. Compared with the water distribution systems developed by Hu Z et al. (2022), this paper can identify more types of false alarms of water quality anomalies and has stronger persistence. It can better identify water quality anomalies caused by accidental factors as pseudo abnormal data (Fig 9), thereby enhancing the feasibility of water quality monitoring and abnormal early warning by linking residual patterns to operationally meaningful anomaly types.

## 5. Conclusions

Based on the problem of false alarms of water quality anomalies in the research of abnormal water quality early warning, this paper proposes a water quality abnormal early warning model from the dual-driven perspective of knowledge and data. In this model, water quality anomalies are first defined and analyzed in detail, and different water quality anomalies are classified into true abnormal data and pseudo abnormal data. Secondly, based on the anomaly analysis, the abnormal data in the training samples is removed. Then, an ensemble learning model for water quality prediction is constructed to improve the prediction accuracy. Finally, the predicted values are compared with the actual values to dynamically determine the abnormal data of water quality and classify them, thereby avoiding false alarms of water quality anomalies as much as possible. By verifying the data of some water quality stations in the Second Phase of the Eastern Route of the South-to-North Water Diversion Project in China, it is found that the water quality anomaly early warning model described in this paper from the knowledge-data-driven perspective can well detect the “true anomaly” in water quality and exclude the false anomalies from the early warning system. This paper combines technology and knowledge, which plays an important role in the application of water quality monitoring and early warning, helping managers better discover the true anomalies in water quality changes, and has greater auxiliary decision-making value. In the future, more types of water quality anomaly determination can be added to the model described in this paper to increase its automatic determination capabilities, and better serve water quality monitoring.

## 6. Limitations and outlooks

Although this paper has significant advantages in identifying water quality anomalies, there are still some research deficiencies. For example, the adaptability of the model to various environments needs to be further tested, and the dynamic update mechanism of the model parameters has not yet been established. There may be a risk of mismatch in the water quality evolution pattern in long-term operation. In the future, research will focus on cross-basin transfer learning, lightweight model reconstruction, and online adaptive optimization.
